# Supplementary material for: Mechanical Properties and Deformation Mechanisms of Graphene Foams with Bi-Modal Sheet Thickness by Coarse-Grained Molecular Dynamics Simulations
Source: Materials (Basel). 2021 Sep 27;14(19):5622. doi: 10.3390/ma14195622 (PMC8509715; doi:10.3390/ma14195622)
Supplement: Supplementary file 1 [file materials-14-05622-s001.zip › materials-1371739-supplementary.pdf]

materials

Supplementary Materials

Mechanical Properties and Deformation Mechanisms of Graphene Foams with Bi-Modal Sheet Thickness by Coarse-Grained Molecular Dynamics Simulations

Shenggui Liu [1], Mindong Lyu [1] and Chao Wang [2,3,*]

[1] School of Mechanics and Civil Engineering, China University of Mining and Technology, Beijing 100083, China; liushg2002@163.com (S.L.); zeqiulmd@126.com (M.L.)
[2] LNM, Institute of Mechanics, Chinese Academy of Sciences, Beijing 100190, China
[3] School of Engineering Science, University of Chinese Academy of Sciences, Beijing 100049, China
* Correspondence: wangchao@lnm.imech.ac.cn; Tel.: +86-10-82543525

Citation: Liu, S.; Lyu, M.; Wang, C. Mechanical Properties and Deformation Mechanisms of Graphene Foams with Bi-Modal Sheet Thickness by Coarse-Grained Molecular Dynamics Simulations. *Materials* **2021**, *14*, 5622. https://doi.org/10.3390/ma14195622

Academic Editor: Liviu Marsavina and Jaroslav Kovacik

Received: 23 August 2021
Accepted: 24 September 2021
Published: 27 September 2021

Publisher's Note: MDPI stays neutral with regard to jurisdictional claims in published maps and institutional affiliations.

Copyright: © 2021 by the authors. Licensee MDPI, Basel, Switzerland. This article is an open access article distributed under the terms and conditions of the Creative Commons Attribution (CC BY) license (https://creativecommons.org/licenses/by/4.0/).

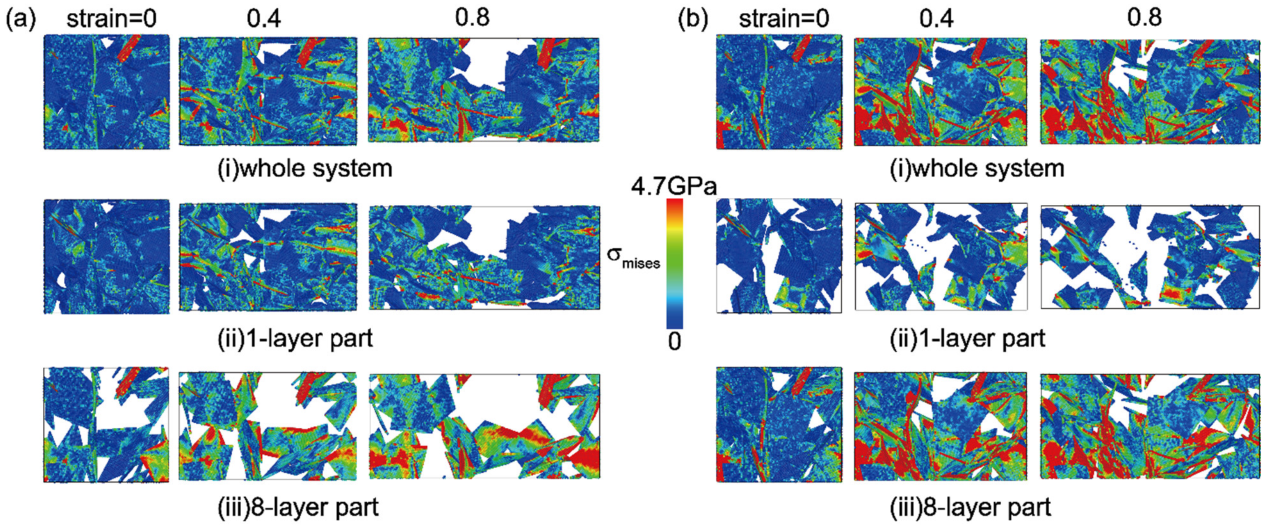

Figure S1. (**a–i**) The distribution of the local von-Mises stress of the whole GrF system with the proportion of 8-layer sheet $v = 30\%$, for a direct comparison of the stress distribution in the 1-layer soft and 8-layer sheets, the two parts are displayed separately in (**a–ii**) and (**a–iii**); (**b–i**) The distribution of the local von-Mises stress of the whole GrF system with the proportion of 8-layer sheet $v = 70\%$, for a direct comparison of the stress distribution in the 1-layer soft and 8-layer sheets, the two parts are displayed separately in (**b–ii**) and (**b–iii**).

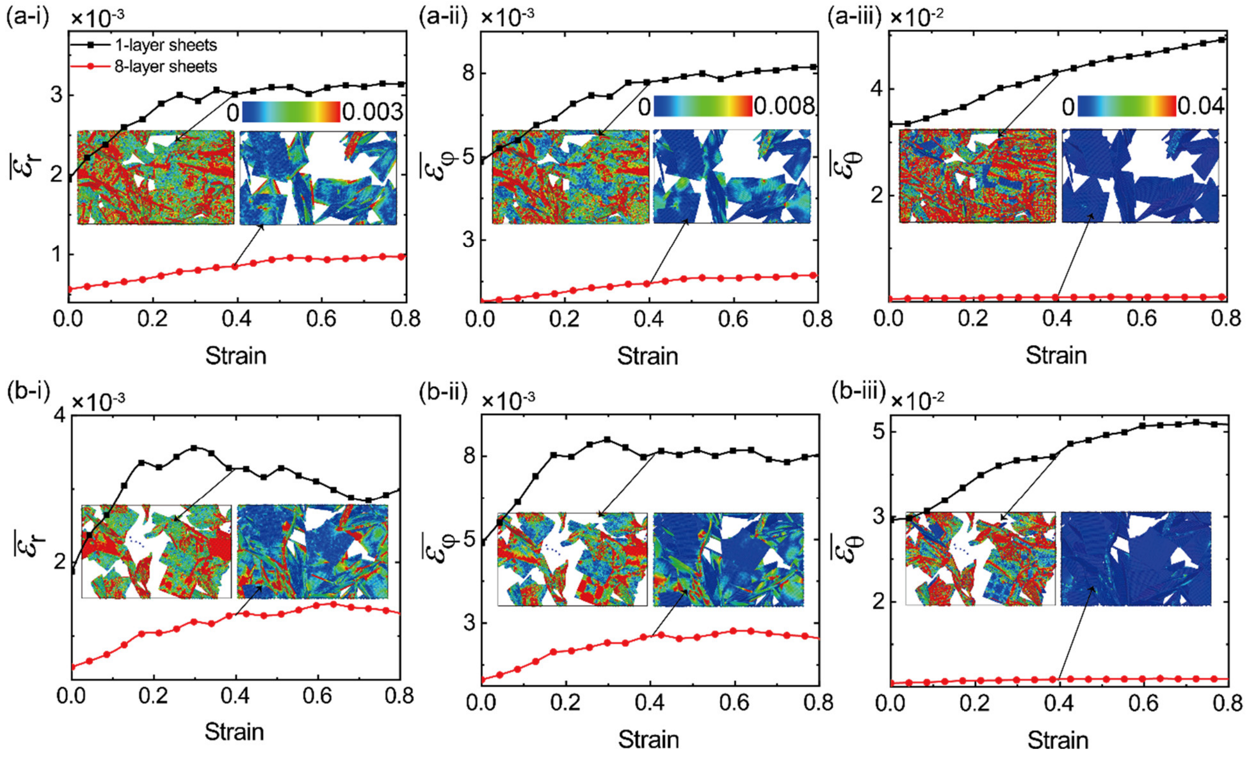

Figure S2. The comparison of the variation of (**a–i**) bond length, (**a–ii**) in-plane shearing angle and (**a–iii**) out-of-plane bending angle in 1-layer soft sheets and 8-layer hard sheets, respectively. The distribution of (**a–i**) the bond strain, (**a–ii**) shear angle, (**a–iii**) bending angle in the whole GrF system with the proportion of 8-layer sheet $v = 30\%$ at the tensile strain of 0.4 for a direct comparison of the strain distribution in 1-layer soft sheets and 8-layer hard sheets, the two parts are displayed separately; The comparison of the variation of (**b–i**) bond length, (**b–ii**) in-plane shearing angle and (**b–iii**) out-of-plane bending angle in 1-layer soft sheets and 8-layer hard sheets, respectively. The distribution of (**b–i**) the bond strain, (**b–ii**) shear angle, (**b–iii**) bending angle in the whole GrF system with the proportion of 8-layer sheet $v = 70\%$ at the tensile strain of 0.4 for a direct comparison of the strain distribution in 1-layer soft sheets and 8-layer hard sheets, the two parts are displayed separately.
